# Effects of Transcranial Direct Current Stimulation on Upper Limb Muscle Strength and Endurance in Healthy Individuals: A Systematic Review and Meta-Analysis

**DOI:** 10.3389/fphys.2022.834397

**Published:** 2022-03-09

**Authors:** Kun Hu, Yu Chen, Feng Guo, Xin Wang

**Affiliations:** College of Human Kinesiology, Shenyang Sport University, Shenyang, China

**Keywords:** transcranial direct current stimulation (tDCS), upper limb, exercise performance, muscle strength, endurance

## Abstract

**Objective:**

Whether transcranial direct current stimulation (tDCS) can improve upper limb muscle strength and endurance in healthy subjects is still controversial. This article reviews the relevant literature on the use of tDCS to improve upper limb muscle strength and endurance in healthy individuals.

**Methods:**

We systematically searched the Cochrane Library, PubMed, EMBASE, and the Web of Science until September 4, 2021. Randomized parallel or crossover experimental studies on the effects of tDCS on upper limb muscle strength and endurance in healthy individuals were included. Review Manager 5.3 software was used to evaluate methodological quality and analyze the combined effect of the included literature.

**Results:**

Twelve studies (189 participants) were included in the qualitative synthesis, and nine studies (146 participants) were included in the meta-analysis. Compared with the control group, the tDCS intervention had no significant effect on improving upper limb muscle strength [I^2^ = 0%, 95% CI (−0.79, 0.23), *p* = 0.98, MD = 0.01]. In this analysis, tDCS had a significant heterogeneity (I^2^ = 87%) in improving upper limb muscle endurance compared with the control group. After the subgroup analysis and the sensitivity analysis, the source of heterogeneity was excluded. The final results showed that tDCS had a significant effect on improving upper limb muscle endurance [I^2^ = 0%, 95% CI (1.91, 4.83), *p* < 0.00001, MD = 3.37].

**Conclusions:**

tDCS has no significant effect on improvement of upper limb muscle strength, but has a significant effect on improving upper limb endurance performance (especially on the non-dominant side).

## Introduction

Exercise performance refers to the collection of the specific physical qualities of the people participating in a certain sport, which is affected by many physical, physiological, and psychological factors, particularly muscle strength and endurance (Hureau et al., 2018; Vaes et al., 2021). Previous studies have generally improved exercise performance by improving muscle strength (Øvretveit and Tøien, 2018; Amara et al., 2021; Ohya et al., 2021) and endurance (Katayama et al., 2019; Wheelock et al., 2020; Jonvik et al., 2021). Ohya et al. (2021) improved the performance of swimmers by enhancing the muscle strength of the inspiratory muscles Katayama et al. (2019) (Ohya et al., 2021) improved running performance by improving respiratory muscle endurance (Katayama et al., 2019). With the development of brain science, researchers have begun to explore the correlation between the two cerebral hemispheres from the perspective of brain mechanisms (Machado et al., 2019). When researchers explored the physiological mechanism of continuous low-intensity maximal isometric voluntary contraction (MIVC) tasks through transcranial magnetic stimulation (TMS), they found that the regulation of the central nervous system was the dominant factor shortening the time of task failure (Søgaard et al., 2006; Klass et al., 2008; Lévénez et al., 2008). This implies that the brain may play a vital role in muscle strength and muscle endurance (Fan and Kayser, 2016; Taylor et al., 2016). However, few techniques improve muscle strength and endurance by modulating the brain function. For example, recognizing that TMS can improve muscle performance (Desmons et al., 2021). However, there are very few brain boosts, such as TMS. Therefore, it is very important to find techniques that engage the brain to enhance muscle strength and muscle endurance. tDCS is a non-invasive brain stimulation technology that induces focal and transient changes in cortical excitability by applying a low-intensity direct current (1–2 mA) to the scalp to regulate neural activities in the cerebral cortex (Nitsche and Paulus, 2001). It has been demonstrated that tDCS is able to alter brain activities and further impact muscle contractions (Nitsche and Paulus, 2000; Stagg and Nitsche, 2011).

At present, although tDCS has been applied in the exercise science field, there is still disagreement about whether tDCS can improve exercise performance. Some studies have shown that tDCS is closely related to muscle strength and muscle endurance, especially of the upper limbs (Lampropoulou and Nowicky, 2013; Krishnan et al., 2014; Abdelmoula et al., 2016; Frazer et al., 2017; Radel et al., 2017; Hikosaka and Aramaki, 2021). In terms of muscle strength, some studies found that tDCS had no effect on upper limb muscle strength (Cogiamanian et al., 2007; Kan et al., 2013; Abdelmoula et al., 2016, 2018). For example, Hikosaka and Aramaki (2021) found that the in the grip movement of 70% MIVC, there was no significant change in MIVC after anodal tDCS (a-tDCS) stimulation by applying 1.5 mA tDCS on the right primary motor cortex (M1) for 15 min. In contrast, some studies found that tDCS can significantly enhance upper limb muscle strength (Lampropoulou and Nowicky, 2013; Williams et al., 2013; Hendy and Kidgell, 2014; Krishnan et al., 2014). In addition, Krishnan et al. (2014) found that MIVC increased significantly after a-tDCS stimulation in 37.5 and 50% MIVC elbow flexion by applying 2 mA to the left M1 for 10 min.

Similarly, in terms of muscle endurance, some studies found that tDCS had no effect on upper limb muscle endurance (Kan et al., 2013; Muthalib et al., 2013; Radel et al., 2017; Abdelmoula et al., 2018). For example, Abdelmoula et al. (2018) found no significant change in time to exhaustion (TTE) after a-tDCS stimulation during thumb abduction at 35% MIVC by applying a 1.5 mA, 10 min tDCS stimulation to the left M1. In contrast, another study found that tDCS could increase the TTE (Cogiamanian et al., 2007; Williams et al., 2013; Abdelmoula et al., 2016). Abdelmoula et al. (2016) also found that a-tDCS stimulation significantly increased the TTE compared to the control group during elbow flexion at 35% MIVC by applying 1.5 mA of tDCS stimulation for 10 min to the left M1.

Therefore, due to the inconsistent results of previous studies, the effect of the tDCS on upper limb muscle strength and endurance is still unclear. In daily life, the improvement of upper limb muscle strength and endurance can support repetitive work, such as work performed by nurses, computer sedentary workers, textile workers, etc. Even in clinical diseases, the improvement of upper limb muscle performance is helpful for patients with lower limb paraplegia and amputation. Previous studies on muscle performance of tDCS mostly focused on the lower limbs (Okano et al., 2015; Lattari et al., 2020) or the entire body (Lattari et al., 2018; Machado et al., 2019), but there have been few studies on the upper limbs alone. This meta-analysis aims to examine the effect of tDCS on upper limb muscle strength and endurance by evaluating and comparing previous research results. It will provide an important theoretical basis for further application in the field of sports science and new evidence for future research.

## Materials and Methods

### Qualified Standards

Inclusion criteria were based on participants, intervention, control, outcome, study design (PICOS).

1. Participants: Healthy adults, both male and female, excluding those with strength training experience, without skeletal or muscle disorders, and without a history of mental illness.
2. Intervention: tDCS (a-tDCS).
3. Control: sham or no tDCS stimulation.
4. Outcome: analysis of upper limb muscle strength-related outcome measures, including MIVC and repetition maximum (RM). Another analysis of upper limb muscle endurance-related outcome measures, included TTE and.
5. Study design: random experiment, parallel or cross study design. Conference abstracts, papers, and book articles were not included.

### Source of Information

As of September 4, 2021, we searched the following databases: Cochrane Library, PubMed, EMBASE, and Web of Science. No filter was applied in this search.

### Search Strategy

The Cochrane library’s search strategy can be found in Supplementary Appendix, which also applies to other databases.

### Select Study

An author (KH) filtered apparent unrelated studies by reading titles and abstracts. Then, the full text of the study was retrieved. According to the inclusion criteria of the study, three authors (KH, YC, and XW) divided it into relevant, possible or irrelevant. The two authors carefully read whether the possible related research conforms to the PICOS principle of this study. We excluded all unrelated studies and carefully judged possible related studies. In cases of different opinions, the third author (FG) finally decided them.

### Data Extraction

For each included article, the data we extracted included the characteristics of the subjects (sample size, age, sex, with or without withdrawal, with or without strength training experience), tDCS intervention programs (electrode size, electrode position, current intensity, and stimulation duration), and main outcomes (muscle strength and endurance of upper limb muscles or muscle groups in isometric exercise and isokinetic exercise). To reduce the risk of bias in the data extraction, the same author twice extracted the data.

### Quality Assessment

The bias risk assessment followed the criteria proposed in the Cochrane Guidelines (Higgins and Green, 2011): (a) random sequence generation; (b) allocation concealment; (c) blinding of participants and personnel; (d) blinding of outcome assessment; (e) incomplete outcome data; (f) selective reporting; and (g) other issues. Two researchers (KH and YC) independently assessed the included studies. According to the criteria defined by Higgins (Higgins and Green, 2011), the assessment questions were divided into low, high and unclear risk biases. If there was disagreement, further discussion was needed, which was decided by a third researcher (FG).

### Quantitative Analysis

Different information was extracted from the selected studies, for example, subject information, selection criteria, intervention protocol, primary outcome, etc., and heterogeneity of the studies was then assessed to determine whether it was appropriate to analyze the synthesis.

A separate meta-analysis was performed for different exercise program types and different tDCS stimulation areas. To explore the differences between the results after the intervention, due to the large differences between the research contents, to eliminate the differences, we chose to use the random effect model. Second, because the measurement results of the data in the extracted studies were the same, we used the mean difference (MD). MD was pooled with a 95% confidence interval (95% CI). Heterogeneity was assessed using chi-squared statistics (Chi^2^) and the heterogeneity index (I^2^). When I^2^ was greater than 50%, there was significant heterogeneity (Higgins and Green, 2011). Subgroup analysis was used to find the source of heterogeneity. Finally, low-quality studies were excluded through sensitivity analysis.

We used RevMan 5.3 for the analysis. All *p* values were analyzed using a two-tailed analysis with a significance level of 5%, except when evaluating heterogeneity between the studies.

## Results

**FIGURE 1:**
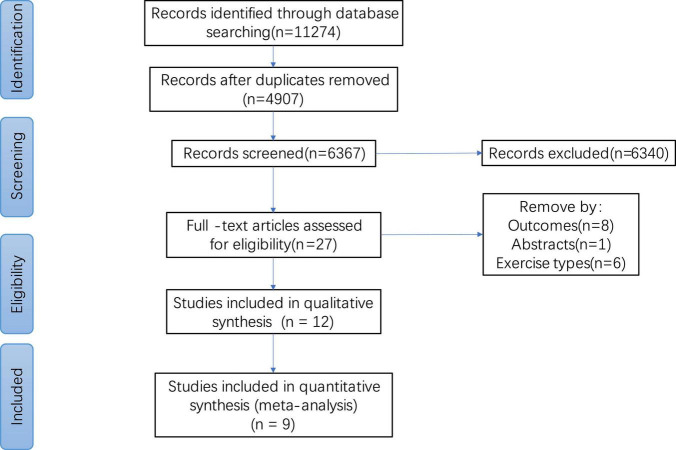
Study flow diagram.

We searched for 1,1274 articles in the Cochrane library, PubMed, EMBASE, and the Web of Science. After removing duplicate articles (*n* = 4907), 6,367 articles were obtained. A total of 6,340 articles were deleted and 27 were retained based on the title and abstract. After full-text browsing, 15 articles were further excluded because they did not meet the inclusion criteria. Twelve articles were included for the qualitative analysis, and nine articles were included for the quantitative analysis. The research flow chart is shown in Figure 1.

### Research Characteristics

Table 1 summarizes the research characteristics of the effect of tDCS on upper limb muscle strength in healthy people. All included studies were randomized, 8 (88.9%) of which were crossover studies and one (11.1%) was a parallel study. A total of 146 participants participated in this study, including 106 males (72.6%) and 40 females (27.4%). The average age of the subjects was between 21.3 ± 0.4 (Radel et al., 2017) and 27.7 ± 8.4 (Kan et al., 2013; Muthalib et al., 2013).

**TABLE 1:** Characteristics of the included studies.

Study information					Participant characteristics		tDCS set-up				
References	Design	Exercise type	Exercise protocol	main outcomes	Gender	Age	Electrode placement	Intensity (mA)	Duration (min)	Density (mA/cm^2^)	Electrode size (cm^2^)
Cogiamanian et al., 2007	Parallel	35%MIVC —	elbow flexion	MIVC TTE*	a-tDCS(9): 5F and 4M control(15): 9F and 6M	24.3	A Right M1 /C Right shoulder	1.5	10	0.043	35
Kan et al., 2013	Cross	35%MIVC —	elbow flexion	MIVC TTE*	a-tDCS(15): M sham(15): M	27.7 ± 8.4	A Right M1 /C Right shoulder	2	10	0.083	24
Williams et al., 2013	Cross	20%MIVC —	elbow flexion	MIVC* TTE*	a-tDCS(18): 9F and 9M sham(18): 9F and 9M	25 ± 6.0	A Right M1 /C Above the left orbit	1.5	20	0.043	35
Abdelmoula et al., 2016	Cross	35%MIVC —	elbow flexion	MIVC TTE*	a-tDCS(11): 3F and 8M sham(11): 3F and 8M	25.0 ± 1.8	A left M1 /C Right shoulder	1.5	10	0.043	35
Hikosaka and Aramaki, 2021	Cross	70%MIVC —	Handgrip	MIVC: RcLa*/RaLc	a-tDCS(21): M sham(21):M	21.7 ± 0.8	A left M1/C right M1	1.5	15	N/D	N/D
Hendy and Kidgell, 2014	Cross	70%1-RM ↑	wrist extension	1-RM strength*	a-tDCS(10): 5F and 5M sham(10): 5F and 5M	25.9 ± 1.4	A Right M1 /C FP1	2	20	0.08	25
Abdelmoula et al., 2018	Cross	35%MIVC —	AOT	MIVC TTE	a-tDCS(10): M sham(10): M	25.5 ± 1.7	A left M1 /C Right shoulder	1.5	10	0.043	35
Radel et al., 2017	Cross	35%MIVC —	elbow flexion	TTE	a-tDCS(22): 9F and13M sham(22): 9F and13M	21.3 ± 0.4	A C2#;A AF4#	2	10	N/D	N/D
Muthalib et al., 2013	Cross	30%MIVC —	elbow flexion	TTE	a-tDCS(15): M sham(15): M	27.7 ± 8.4	A Right M1 /C Right shoulder	2	10	0.083	24

#high-definition transcranial direct current stimulation;*p<0.05; —Isometric strength;↑Dynamic strength; A/C, anode/cathode electrode; F/M, female/male; AOT, abduction of thumb; RM, repetition maximum; M1, primary motor cortex; MIVC, maximal isometric voluntary contraction; TTE, time to exhaustion; tDCS, transcranial direct current stimulation; a-tDCS, anode transcranial direct current stimulation; N/D, not described.

The control group consisted of eight studies (88.9%) and was the sham group (Kan et al., 2013; Muthalib et al., 2013; Williams et al., 2013; Hendy and Kidgell, 2014; Abdelmoula et al., 2016, 2018; Radel et al., 2017; Hikosaka and Aramaki, 2021), and the control group of one study (11.1%) was the non-stimulation group (Cogiamanian et al., 2007). The a-tDCS of five studies (55.6%) was the right M1; the a-tDCS of three studies (33.3%) was the left M1; and the remaining study used high-definition tDCS (HD-tDCS) (Radel et al., 2017). The real stimulation group was also divided into two groups: the C2 group and the AF4 group. The current intensity was 1.5 mA (Cogiamanian et al., 2007; Williams et al., 2013; Abdelmoula et al., 2016, 2018; Hikosaka and Aramaki, 2021) and 2 mA (Kan et al., 2013; Muthalib et al., 2013; Hendy and Kidgell, 2014; Radel et al., 2017), respectively; and the stimulation duration was 10 min (Cogiamanian et al., 2007; Kan et al., 2013; Muthalib et al., 2013; Abdelmoula et al., 2016, 2018; Radel et al., 2017), 15 min (Hikosaka and Aramaki, 2021) and 20 min (Williams et al., 2013; Hendy and Kidgell, 2014), respectively. The electrode size was 24 (Kan et al., 2013; Muthalib et al., 2013) to 35 cm^2^ (Cogiamanian et al., 2007; Williams et al., 2013; Abdelmoula et al., 2016, 2018), and the current density was 0.043 (Cogiamanian et al., 2007; Williams et al., 2013; Abdelmoula et al., 2016, 2018) to 0.083 mA/cm^2^ (Kan et al., 2013; Muthalib et al., 2013). The electrode size and current density of the stimulation electrode in the study conducted by Hikosaka (Hikosaka and Aramaki, 2021) and Radel (Radel et al., 2017) were not known.

The research designs included in this study were all upper limb movements, In all the studies, there were 6 (66.7%) studies involving the elbow joint (Cogiamanian et al., 2007; Kan et al., 2013; Muthalib et al., 2013; Williams et al., 2013; Abdelmoula et al., 2016, 2018; Radel et al., 2017), four studies (Cogiamanian et al., 2007; Kan et al., 2013; Abdelmoula et al., 2016; Radel et al., 2017) included 35% of the MIVC of the elbow joint, two studies included 20% (Williams et al., 2013) and 30% (Muthalib et al., 2013) of the MIVC of the elbow joint; the other three studies (33.3%) involved wrist extension[28], abduction of the thumb[26], and grip exercises (Hikosaka and Aramaki, 2021). In addition, the motion scheme of the eight studies (88.9%) is isometric exercises (Cogiamanian et al., 2007; Kan et al., 2013; Williams et al., 2013; Abdelmoula et al., 2016, 2018; Radel et al., 2017; Hikosaka and Aramaki, 2021); one (11.1%) motion scheme is dynamic strength exercise for 1RM wrist extension (Hendy and Kidgell, 2014).

### Main Results and Quantitative Synthesis

#### tDCS for Improving Muscle Strength of Upper Limb

**FIGURE 2:**
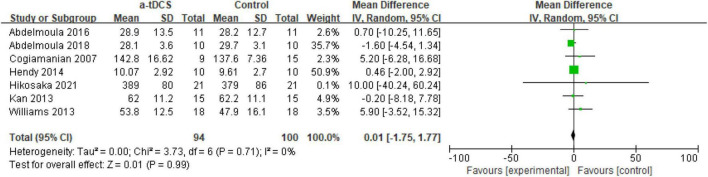
Forest plot for the effect of a-tDCS on enhancing upper limb muscle strength.

Regarding the effect of tDCS on upper limb muscle strength, this meta-analysis included seven studies. They are elbow (4), wrist (1), hand (1), and thumb (1), respectively. Among the outcome indicators of muscle strength, MIVC and 1RM strength are mainly selected as the indicators to evaluate muscle strength. A total of seven research designs were included in the synthesis. Compared with the control group, the application of a-tDCS had no significant effect on improving upper limb muscle strength (*Z* = 0.03, *p* = 0.98). However, the heterogeneity results were good (Chi^2^ = 3.86, *p* = 0.80, I^2^ = 0%), which could be quantitatively synthesized (Figure 2).

Among the four studies (Cogiamanian et al., 2007; Kan et al., 2013; Abdelmoula et al., 2016, 2018), a-tDCS showed no significant difference in the MIVC results after the intervention compared with the control group (*p* > 0.05). Coincidently, most of these studies adopted the same motion design, that is, 35% of the MIVC of the elbow joint (both the dominant side and the non-dominant side). A possible reason is that Cogiamanian (Cogiamanian et al., 2007) was the first researcher to study the effect of tDCS on muscle strength, and the design he used was exactly 35% of the MIVC of the elbow flexion. Therefore, later scholars adopted the research scheme designed by Cogiamanian et al. (2007). In addition, the results of a-tDCS were significantly different (*p* < 0.05) from those of the control group in three studies (Williams et al., 2013; Hendy and Kidgell, 2014; Hikosaka and Aramaki, 2021). Interestingly, in Hikosaka′s study (Hikosaka and Aramaki, 2021), there were two studies designed to exercise 70% handgrip MIVC, with different interventions (stimulation of the left M1 and the right M1, respectively). The results were opposite (compared with the control group, stimulation on the left M1, MIVC showed significant differences; there was no significant difference in MIVC after stimulation on the right M1, and the authors did not report the results after stimulation on the right M1, so we did not include it in this study).

#### tDCS for Improving Muscle Endurance of Upper Limb

**FIGURE 3:**
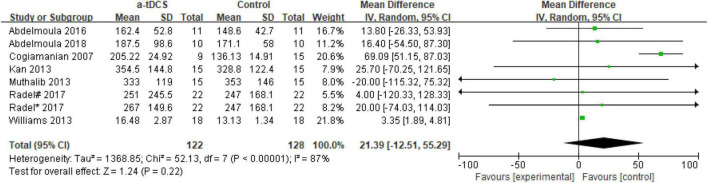
Forest plot for the effect of a-tDCS on enhancing upper limb muscle endurance.

**FIGURE 4:**
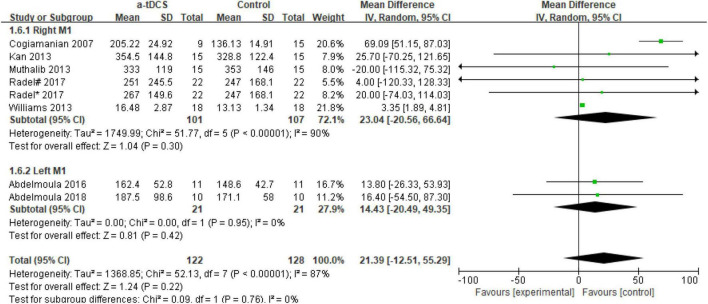
Forest plot of a subgroup analysis of the effect of a-tDCS on enhancing upper limb muscle endurance. Radel#- Stimulate the prefrontal cortex; Radel*- Stimulate the primary motor cortex(M1).

**FIGURE 5:**
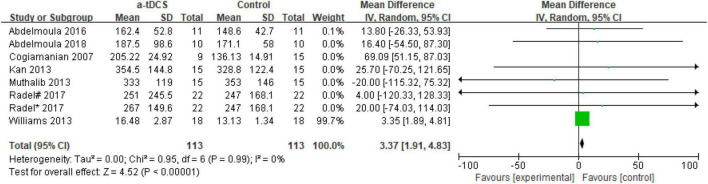
Forest plot of sensitivity analysis of the effect of a-tDCS for enhancing upper limb muscle endurance.

In the studies of the influence of tDCS on upper limb muscle endurance, the TTE is mainly selected as the index to evaluate muscle endurance. A total of eight research designs were included in the synthesis. We found that the results of the a-tDCS intervention did not have a significant indigenous effect (*Z* = 1.24, *p* = 0.22), but there was a significant heterogeneity (Chi^2^ = 52.13, *p* < 0.00001, I^2^ = 87%) (Figure 3), so it could not be quantified and synthesized. Then we tried to perform a subgroup analysis of the TTE and grouped it into the right M1 group and the left M1 group according to the stimulation position of the anode electrode. One study (Radel et al., 2017) showed that the electrode position was on the prefrontal cortex (PFC). Due to the stimulation position on the right side, we merged it into the right M1 group. As shown in Figure 4, the results of the right M1 group showed no significant indigenous effect (*Z* = 1.04, *p* = 0.30), and there was still extremely significant heterogeneity (Chi^2^ = 51.77, *p* < 0.00001, I^2^ = 90%). The results of the stimulation in the left M1 group also had no significant indigenous effect (*Z* = 0.81, *p* = 0.42), but there was no heterogeneity (Chi^2^ = 0.00, *p* = 0.95, I^2^ = 0%). Therefore, we analyzed the sensitivity of the right M1 group and found that when Williams’s study (Williams et al., 2013) was excluded, the heterogeneity changed significantly (Chi^2^ = 5.59, *p* = 0.23, I^2^ = 29%), and the results showed that the a-tDCS intervention had a significant effect (*Z* = 2.20, *p* = 0.03); when Cogiamanian’s study (Cogiamanian et al., 2007) was excluded, heterogeneity decreased directly to 0% (Chi^2^ = 0.95, *p* = 0.99, I^2^ = 0%), and the results also showed significant effects (*Z* = 4.52, *p* < 0.00001). We suspect that there may be sources of risk for heterogeneity in Cogiamanian and Williams studies, and that heterogeneity is higher in Cogiamanian’s studies. The author believes that the heterogeneity in this paper comes from Cogiamanian’s study, which may be the major difference between Cogiamanian and other studies is that the control group of the study is the blank group, rather than the sham group. Therefore, we try to remove Cogiamanian’s research from this study and make quantitative summation again. The results showed that there was no heterogeneity, and tDCS had a significant effect on upper limb muscle strength (Figure 5).

In the five research designs (Kan et al., 2013; Muthalib et al., 2013; Radel et al., 2017; Abdelmoula et al., 2018), a-tDCS had no significant difference in the TTE after the intervention compared with the control group (*p* > 0.05). Interestingly, two of the research designs came from the same study (Radel et al., 2017). Only a-tDCS stimulation sites were different [right prefrontal cortex (PFC) and right M1, respectively], but the results of the TTE showed no significant difference. It is worth noting that in the study of tDCS to improve upper limb muscle endurance, most of the studies chose the elbow joint as the exercise design, and even more, in Cogiamanian, Kan, and Abdelmoula’s study design (Cogiamanian et al., 2007; Kan et al., 2013; Abdelmoula et al., 2016), 35% of the elbow joint MIVC was selected. Although the tDCS stimulation site had left M1 and right M1, the results of the TTE after the intervention were different. This may be related to the different subjects, sample size selection, selection of the control group and other factors.

#### Risk of Bias in Included Studies

**FIGURE 6:**
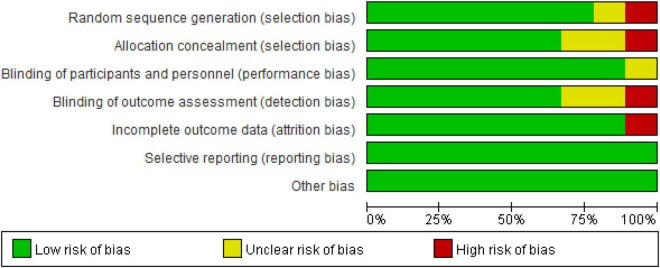
Risk of bias in included studies.

For the risk of bias included in the study as shown in Figure 6, all the studies reported bias (selective reporting results) and other biases were low risk biases. In the random sequence generation and the allocation concealment, one study did not report how to generate random sequences and allocate concealment (Abdelmoula et al., 2016), so it was a high-risk bias. In the implementation of bias (using the blind method for the subjects and the researchers), none of the studies had a high risk of bias. In terms of measurement bias (blinding of the outcome assessments), blinding of outcome assessments in Kan’s study (Kan et al., 2013) is not guaranteed and the results may be affected and assessed as a high-risk bias. In addition, in the follow-up bias (incomplete outcome data), one subject in Radel’s study (Radel et al., 2017) did not complete the experiment, so it was reported as a high-risk bias.

## Discussion

This systematic evaluation and meta-analysis included nine studies that involved 146 subjects in random parallel or crossover experiments to study the effects of tDCS on upper limb muscle strength and endurance in healthy people. In the research scheme, we found that regardless of the control group was a sham or non-stimulated, a-tDCS had no significant effect on upper limb muscle strength. In the study of muscle endurance, we found that a-tDCS had a significant effect on upper limb muscle endurance, especially on the non-dominant side.

A meta-analysis of the effects of tDCS on upper limb muscle strength in this study showed no significant difference between a-tDCS and the control group (Figure 2). Among them, except for Hendy’s study (Hendy and Kidgell, 2014) which is a dynamic strength test, the remainder of the research is on the maximum voluntary isometric contraction. Among these isometric contractions, others are isometric contractions with low motion intensity, ranging from 20 to 35% of MIVC. However, the motor performance and motor significance expressed by these low-intensity motor tasks are actually very limited, and it is difficult to apply these research results to practical conditions. For example, in sports such as rings and judo, these low-intensity experimental results are difficult to apply directly. On the other hand, these designs are due to the limitations of some indoor conditions, and future research should consider the design of different sports intensities in the context of sports. In addition, the size, location, and stimulation scheme of tDCS electrodes in the included studies were inconsistent, which may explain for the diversification of the results. However, at least the evidence in this study does not prove that tDCS can improve isometric muscle strength. In addition, previous studies suggest that tDCS has a “ceiling” effect in improving muscle strength (Kan et al., 2013), that is, the exercise task is too simple, and it is difficult to produce significant effects for stronger individuals. tDCS changes brain neurons by applying a constant weak current (1–2 mA) in the scalp to improve nerve excitability (Nitsche and Paulus, 2000; Polanía et al., 2012). However, the degree of neuronal excitability caused by this weak stimulation is relatively low, so it is difficult to produce an obvious effect for individuals with a high baseline strength (Montenegro et al., 2015). This can explain why previous researchers who used tDCS to study upper limb muscle strength were more inclined to choose a low intensity exercise design, and select several research objectives for the non-dominant side (Lattari et al., 2016; Flood et al., 2017).

The present study showed that only Hendy (Hendy and Kidgell, 2014) explored the effect of tDCS on the dynamic strength of the upper limb (wrist). Compared with the sham group, the strength of wrist 1RM in the a-tDCS group was significantly increased. However, this study is not sufficient to prove that t-DCS is effective in enhancing the dynamic strength of the upper limb muscles. Moreover, in most sports, projects involving isometric muscle strength are far common than those involving dynamic muscle strength, and future research should consider further research on dynamic strength.

Meta-analysis of the effect of tDCS on upper limb muscle endurance in this study showed high heterogeneity (Figure 3), so quantitative synthesis was not possible. In Williams’s study (Williams et al., 2013), compared with the control group, the TTE after a-tDCS intervention increased by approximately 25%. Meanwhile, in Cogiamanian’s study (Cogiamanian et al., 2007), the TTE increased by approximately 50% after a-tDCS intervention compared with the control group, suggesting that a-tDCS can improve muscle endurance. However, the results of these two studies should be treated with caution because there is significant heterogeneity in the synthesis of muscle endurance, and the results of these two studies have slightly uneven weights in the synthesis analysis (21.8 and 20.6%).

We conducted a subgroup analysis to the anode electrode stimulation position. The only two studies in the left M1 group were divided into the 2016 (Abdelmoula et al., 2016) and the 2018 (Abdelmoula et al., 2018) studies conducted by Abdelmoula. In both studies, the stimulation scheme was 1.5 mA a-tDCS stimulation for 10 min with the cathode position on the right shoulder. The exercise intensity was 35% of the MIVC. However, the exercise scheme was different for, namely, elbow flexion and thumb abduction, which contributed to different results. In 2016, the TTE increased by 9.3% after a-tDCS, while in 2018, the TTE did not increase significantly after a-tDCS. Moreover, the quantitative analysis results of the two articles showed that the tDCS intervention did not improve muscle endurance (Figure 4). Although the results of only two studies show that tDCS is ineffective in improving muscle endurance, this suggests that our subsequent studies try to avoid selecting the left M1 as the anode electrode stimulation position. According to the neurocrossover control theory, the left M1 corresponds to the right limb, while the right limb is generally the dominant side. This meta-analysis may explain why tDCS has no significant effect on the muscle endurance of the upper limb on the dominant side of healthy people, which can also explain why an increasing number of scholars choose the non-dominant side as the research goal. This has been confirmed by previous studies. For example, Oki et al. (2016) found that the improvement in endurance level after the a-tDCS intervention was negatively correlated with the baseline maximum strength level, indicating that the smaller the baseline maximum strength, the stronger the effect of tDCS intervention. We also consider that the use of tDCS in improving endurance still exhibits a “ceiling” effect, da Silva Machado et al. (2021) reported that endurance athletes after two bicycle fatigue tasks used traditional tDCS and HD-tDCS intervention for 20 min, with an exercise intensity of 80% peak power and a stimulus intensity of approximately 2 mA. The results did not find that the TTE significantly improved, indicating that the use of tDCS in improving the baseline level of the subjects’ endurance may not be significant.

Regarding the right M1 group, there was still significant heterogeneity after the subgroup analysis. We tried a sensitivity analysis and found that the sources of heterogeneity were in the Cogiamanian and Williams’ studies (Cogiamanian et al., 2007; Williams et al., 2013), and Cogiamanian’s study was the main source of heterogeneity. Therefore, we removed the study and quantified it again. The results showed that tDCS significantly improved upper limb muscle endurance, and there was no heterogeneity (Figure 5). This suggests that tDCS may be beneficial for improving upper limb muscle fatigue on the non-dominant side.

In this meta-analysis, the random effect model was used, and the weight of each study was the variance of the study plus the reciprocal of the variance between the studies. The larger weight may be due to the small variance of the study. At the same time, except for the control group of Cogiamanian’s study (Cogiamanian et al., 2007) which had no stimulation, the control group in the other studies was a sham. In addition, Cogiamanian’s study is the only parallel experimental design, while all the others are cross-experimental designs, which may also be the reason for the large heterogeneity of Cogiamanian’s study (Cogiamanian et al., 2007).

The improvement of upper limb muscle endurance is particularly noteworthy, especially in some high-level athletes, such as rock climbers, kayaking athletes, cyclists, etc. These major competitions may increase the endurance level by 1% and affect the direction of the entire competition. This study only enrolled normal healthy adults, whereas people with training experience, such as athletes, were excluded. The reason is that there are few articles that study that impact of tDCS on the upper limb endurance of athletes, and there are important differences in that research methods, participants and intervention programs. At the same time, before the safety of tDCS is fully evaluated, it remains to be discussed when it is applicable to high-level athletes and even healthy people as a conventional means.

### Limitation

At present, the use of tDCS to improve upper limb muscle strength and endurance remains controversial. Based on this meta-analysis, we summarize the following limitations: (1) there is no standardized and unified design for the various methodological variables (electrode position, stimulation time, stimulation intensity, etc.) included in this study, which will lead to a high degree of variability in the results of various studies and more uncertainty for the quantitative analysis and synthesis results. However, there is no solution to this situation, which may require scholars to conduct studies with a more precise experimental design to systematically standardize this problem in the future. (2) There is no consensus on the effects of tDCS on upper limb motor performance, and the quantitative analysis samples included in this meta-analysis were too few, which would lead to the possibility of false negative or false-positive results in our synthesis. (3) In the study of upper limb endurance, only two articles (Abdelmoula et al., 2016, 2018) focused on the dominant side. Although the results showed that tDCS had no significant effect on improving the muscle endurance of the dominant side of the upper limb, we were cautious about this result due to the lack of samples. (4) This study has not reported on the safety (structural or functional damage to the human body) and sensitivity (discomfort but no damage to the human structure or function, such as itching and pain) (Woods et al., 2016) of tDCS. Although a recent study has shown that up to 4 mA tDCS is safe, well tolerated and will not cause any serious adverse reactions (Nitsche and Bikson, 2017), we should be careful about the application of tDCS in truly healthy people and professional athletes. (5) Five of the nine studies included in this paper included different sex groups, but none of them discussed sex disaggregation, which is a limitation of this paper. Since the tDCS effects on males and females are completely different, the acceptable intensity of the current for males is higher than for females (Russell et al., 2014), but the stimulation effect on females is better than that on males (Boggio et al., 2008; de Tommaso et al., 2014). Future tDCS studies should focus on the interference factor of gender. If different sex participants are included, then sex should be discussed in the groups. (6) Some researchers believe that the improvement in motor performance after tDCS intervention may be the result of corticospinal excitability or other brain regulation (Angius et al., 2018). Only a small portion of the studies included in this paper detected neural activity during or after tDCS, and the specific physiological mechanism by which tDCS improves exercise performance is still unknown. Future research should combine tDCS technology with neuroimaging technology, such as functional magnetic resonance imaging (fMRI) (Grami et al., 2021; Kurtin et al., 2021), electroencephalography (EEG) (Miraglia et al., 2021; Xie et al., 2021), functional near-infrared spectroscopy (fNIRS) (Muthalib et al., 2018; Besson et al., 2019), and positron emission tomography (PET) (Rudroff et al., 2020; Bunai et al., 2021), to record brain nerve activity in real time, and establish the relationship between nerve activity and exercise performance.

## Conclusion

This systematic review and meta-analysis found that tDCS did not improve upper limb muscle strength, but had a significant effect on improving upper limb muscle endurance (non-dominant side). tDCS can be used as an auxiliary tool to improve upper limb muscle endurance, especially in exercise involving upper limb isometric contraction.

## Data Availability Statement

The original contributions presented in the study are included in the article/Supplementary Material, further inquiries can be directed to the corresponding author/s.

## Author Contributions

KH, FG, and XW designed the study. KH and YC performed the data extraction. XW polished the language. KH and FG wrote the manuscript. All authors commented on the manuscript.

## Conflict of Interest

The authors declare that the research was conducted in the absence of any commercial or financial relationships that could be construed as a potential conflict of interest.

## Publisher’s Note

All claims expressed in this article are solely those of the authors and do not necessarily represent those of their affiliated organizations, or those of the publisher, the editors and the reviewers. Any product that may be evaluated in this article, or claim that may be made by its manufacturer, is not guaranteed or endorsed by the publisher.

## Abbreviations

tDCS: transcranial direct current stimulation
a-tDCS: anodal transcranial direct current stimulation
MIVC: maximal isometric voluntary contraction
TMS: transcranial magnetic stimulation
TTE: time to exhaustion
M1: primary motor cortex
MD: mean difference
RM: repetition maximum
fMRI: Functional Magnetic Resonance Imaging
EEG: Electroencephalogram
fNIRS: functional Near-infrared Spectroscopy
PET: Positron Emission Tomography.
