# Supplementary material for: Effects of Transcranial Direct Current Stimulation on Upper Limb Muscle Strength and Endurance in Healthy Individuals: A Systematic Review and Meta-Analysis
Source: Front Physiol. 2022 Mar 9;13:834397. doi: 10.3389/fphys.2022.834397 (PMC8959826; doi:10.3389/fphys.2022.834397)
Supplement: Supplementary file 1 [file Table_1.DOCX]

**Appendix-** **Search strategy**

#1 MeSH descriptor: [Transcranial Direct Current Stimulation] explode all trees

#2 (Cathodal Stimulation Transcranial Direct Current Stimulation):ti,ab,kw OR (Cathodal Stimulation tDCS):ti,ab,kw OR (Anodal Stimulation Transcranial Direct Current Stimulation):ti,ab,kw OR (Anodal Stimulation tDCS):ti,ab,kw OR (HD-tDCS):ti,ab,kw (Word variations have been searched)

#3 #1 or #2

#4 (performance):ti,ab,kw OR (activity):ti,ab,kw OR (fatigue):ti,ab,kw OR ("muscle strength"):ti,ab,kw OR (endurance):ti,ab,kw (Word variations have been searched)

#5 #3 and #4
